# Changes in Self‐Reported Excessive Daytime Sleepiness Are Associated With 5‐Year All‐Cause Mortality Risk Among Veterans

**DOI:** 10.1111/jsr.70168

**Published:** 2025-08-19

**Authors:** Katherine G. Bay, Arash Maghsoudi, Amin Ramezani, Drew A. Helmer, Amir Sharafkhaneh, Javad Razjouyan

**Affiliations:** ^1^ Center for Innovations in Quality, Effectiveness and Safety (IQuESt) Houston Texas USA; ^2^ Department of Medicine Baylor College of Medicine Houston Texas USA; ^3^ Big Data Scientist Training Enhancement Program (BD‐STEP), VA Office of Research and Development Washington DC USA; ^4^ Michael E. DeBakey VA Medical Center Houston Texas USA; ^5^ Brigham and Women's Hospital, Harvard Medical School Boston Massachusetts USA

**Keywords:** all‐cause mortality, Epworth sleepiness scale, excessive daytime sleepiness, natural language processing

## Abstract

Excessive daytime sleepiness (EDS) is linked to adverse clinical outcomes. This study evaluated changes in a validated tool to assess EDS, the Epworth Sleepiness Scale (ESS) and mortality risk. This retrospective cohort study included Veterans receiving sleep‐related services in the Department of Veterans Affairs (VA) from October 4, 1999 to August 18, 2018, with two qualifying ESS measures. ESS values were extracted from patient notes using a validated natural language processing (NLP) pipeline (96% accuracy). ESS scores were categorised as Normal (0–10) or Abnormal (11–24). Patients were grouped based on ESS changes: Normal‐Normal, Normal‐Abnormal, Abnormal‐Abnormal and Abnormal‐Normal. Cox proportional hazards models adjusted for time, age, sex, race and comorbid conditions assessed the risk of 5‐year all‐cause mortality. Among 17,967 qualifying Veterans (mean age: 56.3 (SD 13.5) years), 11.75% died within 5 years of the second ESS measure. At baseline, 9342 (52.0%) had EDS, for whom 2232 (12.4%) improved to normal by the second exam (Abnormal‐Normal). The Normal‐Abnormal group had a 25% higher adjusted all‐cause mortality risk within 5 years (aHR: 1.25, 95% CI: 1.09, 1.44) compared to the Normal‐Normal group, with progressively increasing risk after age 55. In contrast, neither persistent abnormal sleepiness (Abnormal‐Abnormal) nor improvement from abnormal to normal (Abnormal‐Normal) was associated with significantly different mortality risk compared to the Normal‐Normal group. ESS can efficiently identify EDS, which may serve as a clinical marker for 5‐year all‐cause mortality risk, particularly among Veterans seeking VHA sleep services aged 55 and older.

## Graphical abstract

Natural Language Processing (NLP) was used to identify 17,967 Veterans with a validated measure of excessive daytime sleepiness (EDS) at an initial exam and an index exam. Veterans who converted from a Normal to Abnormal ESS had a 25% greater risk of 5‐year all‐cause mortality compared to Normal‐Normal, after adjustment.

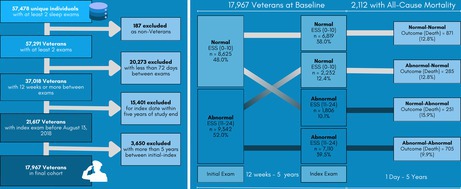

## 1 Introduction

Over 30% of American adults suffer from a form of sleep deficiency such as insufficient sleep, irregular timing of sleep or poor sleep quality (Kolla et al. 2020; National Heart, Lung, and Blood Institute 2019). Sleep deficiency may lead to excessive daytime sleepiness (EDS), which often worsens with age and is associated with a range of comorbidities including cardiovascular disease, obstetric sleep apnea, psychiatric disorders and obesity (Liew and Aung 2021; Fernandez‐Mendoza et al. 2015). EDS symptoms vary widely, ranging from feelings of excessive sleepiness to falling asleep without prodromal drowsiness (Gandhi et al. 2021). Critically, EDS diminishes attentiveness, increases the risk of accidents, and has been linked to an elevated risk of mortality over time (Hublin et al. 2007).

Military Veterans are particularly susceptible to sleep disorders, often due to unique service‐connected challenges such as deployment‐related stress, post‐traumatic stress disorder (PTSD) and traumatic brain injuries. Veterans experience a higher prevalence of sleep disturbances compared to the general population (The Management of Chronic Insomnia Disorder and Obstructive Sleep Apnea Work Group 2019). Previous studies estimate over 20% of Veterans get insufficient sleep, often co‐occurring with other comorbid conditions more prevalent among Veterans and older adults (Faestel et al. 2013; Ocasio‐Tascón et al. 2006).

The Veterans Health Administration (VHA) recognises the high impact of sleep disorders on Veteran health and wellbeing. Consequently, the VHA has prioritised the integration of sleep medicine services into Veterans clinical care, including routinely collecting measures of perceived sleepiness (The Management of Chronic Insomnia Disorder and Obstructive Sleep Apnea Work Group 2019). The Epworth Sleepiness Scale (ESS) is a validated questionnaire used to capture self‐reported EDS by characterising an individual's likelihood of falling asleep in various daily situations (Johns 1991; Martin et al. 2020). The clinical value of this 8‐item survey lies within its ability to help clinicians parsimoniously dichotomise patients into normal (scores 0–10) or abnormal (11–24) EDS during a clinic visit. Moreover, this brief survey has been collected at each VA sleep clinic visit and stored within the VA electronic health record (EHR) for decades, but has remained ‘buried’ as unstructured, free‐form text, which has made it historically difficult to extract at scale (National Heart, Lung, and Blood Institute 2019; Faestel et al. 2013). Therefore, it has been difficult to characterise the clinical impact of longitudinal changes in ESS categories (normal vs. abnormal) over time within the VHA (Maghsoudi et al. 2024).

In this study, we utilised a natural language processing (NLP) pipeline to retrospectively extract over a hundred thousand ESS measures of excessive daytime sleepiness from free‐form texts. We evaluated the dynamic relationship between ESS at sequential sleep clinic visits to characterise 5‐year, all‐cause mortality risk among Veterans from October 1999 to August 2018, particularly in the context of age. We hypothesised that Veterans with ESS changes from normal at baseline to abnormal at the next sleep clinic visit were at elevated risk of all‐cause mortality compared to those with normal ESS at both exams.

## 2 Methods

This study was approved with a waiver of informed consent and HIPAA authorization by Baylor College of Medicine IRB (H‐35366); VA R&D; and Michael E. DeBakey Veteran Affairs Medical Center research and development committee approved this research.

### 2.1 Study Population

**FIGURE 1:**
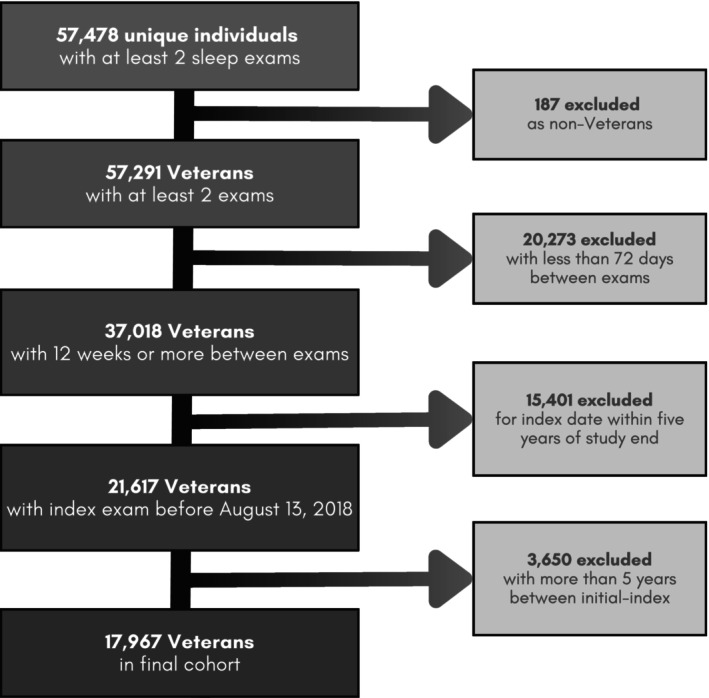
Flowchart of Veteran inclusion. The selection criteria used in the final cohort of 17,967 Veterans evaluated in this study. Participants were only included if they were Veterans, had at least 12 weeks and less than 5 years between initial and index exam before August 13, 2018, allowing for a 5‐year observation for the outcome of interest (all‐cause mortality).

**FIGURE 2:**
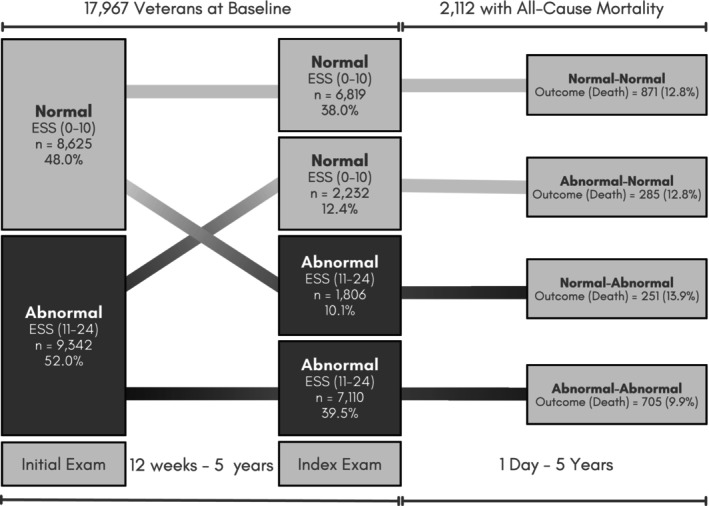
ESS characterisation and outcome status across the study period. Categorisation of the participants by Epworth Sleep Scale (ESS) changes over time. Veterans were observed for up to 5 years for the outcome of interest, all‐cause mortality.

A cohort of Veterans in the VHA was retrospectively identified as having any sleep diagnosis or sleep‐related services from October 4, 1999 to August 18, 2018, using the VHA Corporate Data Warehouse (CDW) (Maghsoudi et al. 2024). The CDW houses clinical and administrative data from the entire national VHA system. The VHA provides care to U.S. Veterans who may qualify for VA Health Care based on their history of military service, level of ‘service connectedness’ and financial status (Veterans Affairs 2024). Inclusion criteria were restricted to Veterans with two or more ESS values at least 12 weeks apart, consistent with clinically meaningful differences between ESS values reported by Rosenberg et al., and no more than 5 years apart (Rosenberg et al. 2022). A flowchart of Veteran inclusion in this cohort is detailed in Figure 1. The 5‐year maximum was chosen after iteratively testing model stability across each year difference from 1 to 10, such that 5 was determined to maximise predictive power and yield clinically meaningful differences over time. The exam closest in time to death or the study end date was predominantly classified as the ‘index’ exam, with the exam directly preceding designated as the ‘initial’ exam (Figure 2).

Approximately 2.5% (*n* = 448) of Veterans had two exams less than 12 weeks apart with a third, earlier qualifying exam prior to the initial exam. A sensitivity analysis concluded that the potential exposures and outcomes of these Veterans did not differ significantly from the rest of the cohort; the initial exam was designated to this earlier time point.

### 2.2 Variables of Interest

*Primary Exposure*: Excessive daytime sleepiness. The ESS is a self‐administered questionnaire that produces a subjective measure of EDS ranging from 0 to 24. The respondent characterises their sleepiness based on ‘no‐’ (0), ‘slight‐’ (1), ‘moderate‐’ (2) or ‘high‐’ (3) ‘chance of dozing’ during eight different daily scenarios. As per Johns et al., categorisation of sleepiness levels are traditionally used, such that a total ESS of 0–10 is considered ‘normal’ and ESS of 11–24 is considered excessively sleepy or abnormal (Johns 1991). Despite ESS being routinely administered during VHA sleep clinic encounters, these measures existed in the electronic health record as free‐form text and historically required tedious chart review to consolidate scores from provider notes. However, the present study leveraged a validated natural language processing (NLP) pipeline to capture ESS values from sleep‐related free‐form texts validated in this cohort with 96% accuracy as detailed by Maghsoudi et al. (2024).

For the present study, patients were categorised according to ESS change state from their initial to index exams: Normal‐Normal, Normal‐Abnormal, Abnormal‐Abnormal and Abnormal‐Normal (Figure 1).

*Primary Outcome*: Five‐year all‐cause mortality. All‐cause mortality was determined by linking to the National Death Index through August 18, 2023 (National Center for Health Statistics 2024). Participants who died within 5 years of their index exam were classified as having the outcome of interest. All others were censored at the end of the 5‐year follow‐up period.

*Covariates*: Sociodemographic factors including sex, age, race, ethnicity and smoking status were obtained from patient clinical data. Races represented included Black or African American (18.85%), White or Caucasian (74.17%) and ‘All Other’, which included American Indian or Alaska Native (0.90%), Asian (0.78%), Native Hawaiian or Other Pacific Islander (0.81%), and those who answered ‘Unknown’ or declined to answer (4.49%). Smoking status was categorised as ‘ever smoker’, those reporting former or current smoking status and ‘never smoker’; however, this variable had high missingness at both time points and was not included in the final model due to potential for bias due to missingness (87%–88%).

Body mass index (BMI) was calculated at each exam and categorised using traditional cut‐points (Nuttall 2015). Charlson‐Deyo Comorbidity Index (CCI) is a single score derived from the sum of 17 disease categories based on International Classification of Diseases (ICD) codes, each weighted based on mortality risk (Charlson et al. 1987; Deyo et al. 1992). Unique comorbid diagnoses available at both exams were also separately evaluated to explore potential relationships with incident comorbid conditions and ESS change. For descriptive tables, severity of CCI was dichotomised using traditional cut‐points as mild (0–2) or moderate‐high (> 2) (Huang et al. 2014). Patients with PTSD, depression or Obstructive Sleep Apnea (OSA) diagnoses on or prior to the initial exam were identified based on ICD‐9 and ICD‐10 codes in the clinical records. OSA diagnoses occurring after a qualifying exam were not attributed post hoc to minimise temporal bias. Age, BMI and CCI at the index exam were evaluated as a continuous variable for model building.

Variation in follow‐up time between the initial and index exams initially spanned over 22 years. This time difference had minimal impact on model stability for up to 5 years, which was approximately twice the standard deviation of mean time between exams (1023.2 days). Therefore, Veterans with initial and index exams spanning more than 5 years (1825 days) apart were excluded, and time between exams was included as a continuous variable in model building. Comparative models were performed with participants stratified by intervals of follow‐up time from initial to index exams (1 year, 2 years, 3 years, 4 years and 5 years).

### 2.3 Statistical Analysis

Baseline characteristics at the initial exam were evaluated using mean and standard deviations for continuous variables and counts and percentages for categorical variables. Univariable relationships for demographic characteristics with ESS change status were evaluated using chi‐squared tests for categorical variables and Kruskal‐Wallis tests for continuous variables. The odds of Normal‐Abnormal ESS change status co‐occurring with the incidence of each of the 17 CCI component diseases (absent at initial exam and present at index exam) and BMI increasing from below 35 kg/m^2^ to over 35 kg/m^2^ were also evaluated (Winter et al. 2014). Bonferroni correction for multiple testing was employed at *α* = 0.05 to yield a two‐sided significance value of 0.003.

Cox proportional hazards models were used to calculate hazard ratios (HR) with 95% confidence intervals (CI) using STATA 17 (StataCorp, College Station, Texas). Purposeful variable selection was utilised to incorporate variables that met preliminary criteria (*p* < 0.20) and their interaction terms into a preliminary model to evaluate predictors of mortality. All time‐varying predictors considered were analysed from the time of the index exam. Covariates and interaction terms deemed biologically plausible and yielding a likelihood ratio *p*‐value of 0.10 were included if they improved model fit overall. Diagnostics were conducted to ensure model assumptions were met. Harrell's C‐index was used to assess the predictive ability of the final model. All analyses were performed, and a two‐sided probability value < 0.05 was considered statistically significant.

## 3 Results

### 3.1 Sample Characteristics

The study population consisted of 17,967 Veterans with two sleep clinic encounters at least 12 weeks and no more than 5 years apart (Figure 1). Most were non‐Hispanic, white and male, with 62.6% over the age of 55 years (Table 1). At the initial exam, nearly two‐thirds of Veterans had a BMI over 30 kg/m^2^ (*n* = 11,719) and 12,541 (70%) had a CCI below 2. The mean time between initial and index exam was 1.28 years (SD 1.31) with a median time of 7.4 months. Nearly 90% of the study population (*n* = 15,855) survived and were censored at the end of the 5‐year follow‐up period.

**TABLE 1:** Baseline characteristics at initial exam for Veteran cohort from October 4, 1999 to August 18, 2018 (*N* = 17,967).

Categorical variables	*N*	%
ESS at initial visit		
Normal (≤ 10)	8625	48.0%
Abnormal (11–24)	9342	52.0%
Sex		
Male	16,657	92.7%
Female	1310	7.3%
Age category at initial visit		
18– < 30	776	4.3%
30– < 40	1845	10.3%
40– < 50	2788	15.5%
50– < 60	4341	24.2%
60– < 70	5863	32.6%
70– < 80	1961	10.9%
≥ 80	393	2.2%
Race		
White	13,326	74.2%
Black	3386	18.9%
Other*	1255	7.0%
Ethnicity		
Not Hispanic/Latino	16,997	94.6%
Hispanic/Latino	970	5.4%
Smoking status		
Ever smoker	1391	3.6%
Non‐smoker	637	7.7%
No response	15,939	88.7%
CCI at initial visit		
0–1	12,541	69.8%
≥ 2	5426	30.2%
Other comorbidities		
Obstructive sleep apnea*	2891	16.1%
Depression	868	4.8%
PTSD	746	4.2%
BMI at initial visit		
< 18.5 (underweight)	32	0.2%
18.5– < 25 (normal)	1250	7.0%
25– < 30 (overweight)	4966	27.6%
30– < 35 (obese I)	5894	32.8%
35– < 40 (obese II)	3542	19.7%
> 40 (obese III)	2283	12.7%

Continuous variables	Mean	SD
Age at initial visit (years)	56.3	13.5
BMI at initial visit (kg/m^2^)	33.0	6.2
CCI at initial visit (0–17)	1.3	1.8

* Includes comorbid insomnia and sleep apnea (COMISA) diagnoses.

### 3.2 Excessive Daytime Sleepiness

Although 9342 (52.0%) of the total cohort (*N* = 17,697) had an abnormal ESS at their initial exam, 2232 (23.9%) returned to normal by their index exam (Figure 2). In contrast, 7110 (39.5%) had an abnormal ESS at both initial and index exams (Abnormal‐Abnormal) and 1806 (10.1%) converted from normal‐abnormal.

The distribution of ESS change categories varied across some subpopulations. Among all female participants (*n* = 1310), nearly half (48.6%) had Abnormal‐Abnormal ESS (*n* = 636), whereas only 38.9% of males were Abnormal‐Abnormal (6474/16,657) (Table 2, *p* < 0.001). However, 28.6% of females were Normal‐Normal (374/1310) compared to 38.7% of males (6445/16,657) (Table 2, *p* < 0.001). Variation also existed across different races and ethnicities. Among the 3386 total Veterans identifying as black, 48.2% were Abnormal‐Abnormal (*n* = 1631), compared to 37.2% of white Veterans (4959/13,326) and 41.4% Other (520/1255) (Table 2, *p* < 0.001). Those who identified as Hispanic/Latino (*n* = 970) had a higher proportion of Normal‐Abnormal ESS (118/970) compared to those who were not Hispanic/Latino (1688/16,997) (Table 2; *p* = 0.024).

**TABLE 2:** Demographic characteristics at index exam by ESS change status (*N* = 17,967).

Categorical variables	*N* (%)	Normal‐Normal *n* = 6819 (37.95%)	Normal‐Abnormal *n* = 1806 (10.05%)	Abnormal‐Abnormal *n* = 7110 (39.57%)	Abnormal‐Normal *n* = 2232 (12.42%)
		*N* (%)	*N* (%)	*N* (%)	*N* (%)
Deaths	2112 (11.75%)	871 (12.77%)	251 (13.9%)	705 (9.92%)	285 (12.77%)
Sex					
Male	16,657 (92.71%)	6445 (94.52%)	1671 (92.52%)	6474 (91.05%)	2067 (92.61%)
Female	1310 (7.29%)	374 (5.48%)	135 (7.48%)	636 (8.95%)	165 (7.39%)
Age (Cat) at index visit					
≥ 55	11,244 (62.58%)	4852 (71.15%)	1100 (60.91%)	3832 (53.9%)	1460 (65.41%)
Race					
White	13,326 (74.17%)	5417 (79.44%)	1334 (73.86%)	4959 (69.75%)	1616 (72.4%)
Black	3386 (18.85%)	978 (14.34%)	332 (18.38%)	1631 (22.94%)	445 (19.94%)
Other*	1255 (6.99%)	424 (6.22%)	140 (7.75%)	520 (7.31%)	171 (7.66%)
Ethnicity					
Hispanic/Latino	970 (5.4%)	313 (4.59%)	118 (6.53%)	415 (5.84%)	124 (5.56%)
Smoking status					
Ever smoker	1598 (8.9%)	663 (9.7%)	137 (7.6%)	620 (8.7%)	178 (8.0%)
Non‐smoker	740 (4.1%)	284 (4.2%)	74 (4.1%)	308 (4.3%)	74 (3.3%)
No response	15,629 (87.0%)	5872 (86.1%)	1595 (88.3%)	6182 (87.0%)	5872 (86.1%)
CCI at index visit					
≥ 2	6101 (33.96%)	2486 (36.46%)	637 (35.27%)	2188 (30.77%)	790 (35.39%)
Depression at initial visit	868 (4.83%)	332 (4.87%)	77 (4.26%)	364 (5.11%)	95 (4.26%)
PTSD at initial visit	746 (4.15%)	257 (3.77%)	66 (3.65%)	335 (4.71%)	88 (3.94%)
OSA at index visit*	3347 (18.6%)	1340 (19.7%)	310 (17.2%)	1316 (18.5%)	381 (17.1%)
BMI at index visit					
< 18.5 (underweight)	32 (0.18%)	11 (0.16%)	4 (0.22%)	11 (0.15%)	6 (0.27%)
18.5– < 25 (normal)	1198 (6.67%)	527 (7.73%)	122 (6.76%)	405 (5.7%)	144 (6.45%)
25– < 30 (overweight)	4847 (26.98%)	1938 (28.42%)	467 (25.86%)	1868 (26.27%)	574 (25.72%)
30– < 35 (obese I)	5893 (32.8%)	2212 (32.44%)	577 (31.95%)	2369 (33.32%)	735 (32.93%)
35– < 40 (obese II)	3604 (20.06%)	1293 (18.96%)	378 (20.93%)	1481 (20.83%)	452 (20.25%)
> 40 (obese III)	2393 (13.32%)	838 (12.29%)	258 (14.29%)	976 (13.73%)	321 (14.38%)

Continuous variables	Mean (SD)	Mean (SD)	Mean (SD)	Mean (SD)	Mean (SD)
Age at index visit	57.53 (13.49)	60.20 (13.00)	58.10 (13.00)	54.90 (13.50)	57.20 (14.00)
BMI at index visit	33.08 (6.25)	32.70 (6.20)	33.30 (6.40)	33.30 (6.30)	33.30 (6.20)
CCI at index visit	1.48 (1.95)	1.50 (1.90)	1.60 (2.10)	1.40 (1.90)	1.50 (2.00)
Follow‐up time (days)	468.80 (479.7)	435.62 (464.60)	571.97 (515.86)	446.69 (467.22)	553.93 (510.18)
Time to death (index‐death)	961.98 (505.01)	968.90 (503.60)	970.90 (502.00)	950.20 (518.40)	960.80 (477.00)

*Note*: Demographic characteristics of participants at index exam by ESS change status.

* Includes COMISA diagnoses.

### 3.3 Five‐Year Mortality

Nearly 12% of participants (*n* = 2112) died of any cause within 5 years of their index exam (Figure 2). On average, those who died were 67.46 (SD: 10.20) years old at their index exam with a CCI of 3.31 (SD: 2.63). Among those who died, most were male, non‐Hispanic, and white. Compared to older Veterans who died, those younger than 55 had a higher proportion of females (10.05% vs. 1.99%, respectively) and those identifying as Hispanic/Latino (6.0% vs. 2.7%, respectively). Older Veterans who died also had a higher proportion with a CCI of 2 or more (73.3% vs. 42.2%). Among those who died, the average time to death after the date of the index exam was 961.98 days (2 years and 8 months) (Table 2).

Purposeful variable selection was used to identify age, sex, CCI and race as potential confounders in the relationship between 5‐year all‐cause mortality and ESS change status and were included in the final model. Follow‐up time and prior diagnoses of depression, PTSD and OSA were also included to minimise residual confounding (Table 3; Harrell's C Index = 0.785). After adjustment, the risk of all‐cause mortality within 5 years of the index exam was 25% higher among Veterans with a Normal‐Abnormal ESS compared to those with Normal‐Normal ESS (aHR: 1.25; 95% CI: 1.09, 1.44; *p* = 0.002). Unexpectedly, persistent abnormal sleepiness (Abnormal‐Abnormal) was not associated with higher adjusted risk compared to Normal‐Normal (aHR: 1.02; 95% CI: 0.93–1.13, *p* = 0.648). Similarly, those who improved from Abnormal‐Normal ESS did not have a significant reduction in 5‐year all‐cause mortality risk compared to Normal‐Normal (aHR: 1.04; 95% CI: 0.91–1.19; *p* = 0.547).

**TABLE 3:** Adjusted 5‐year all‐cause mortality risk by ESS status at index exam and by ESS change over time (crude and adjusted).

Categorical variables	*N* (%)	Crude HR (95% CI)	*p*	aHR* (95% CI)	*p*
ESS at index exam					
Normal (≤ 10)	9051 (50.38%)	Ref		Ref	
Abnormal (11–24)	8916 (49.62%)	0.83 (0.76, 0.91)	< 0.001	1.07 (0.98, 1.16)	0.154
ESS change category					
Normal‐Normal	6819 (37.95%)	Ref		Ref	
Normal‐Abnormal	1806 (10.05%)	1.10 (0.95, 1.26)	0.201	1.25 (1.09, 1.44)	0.002
Abnormal‐Abnormal	7110 (39.57%)	0.77 (0.69, 0.85)	< 0.001	1.02 (0.93, 1.13)	0.648
Abnormal‐Normal	2232 (12.42%)	1.00 (0.88, 1.14)	0.995	1.04 (0.91, 1.19)	0.547

* Adjusted for time between initial and index exams (days), age, sex, race, current CCI, history of OSA, PTSD and depression; Harrell's C = 0.785.

**FIGURE 3:**
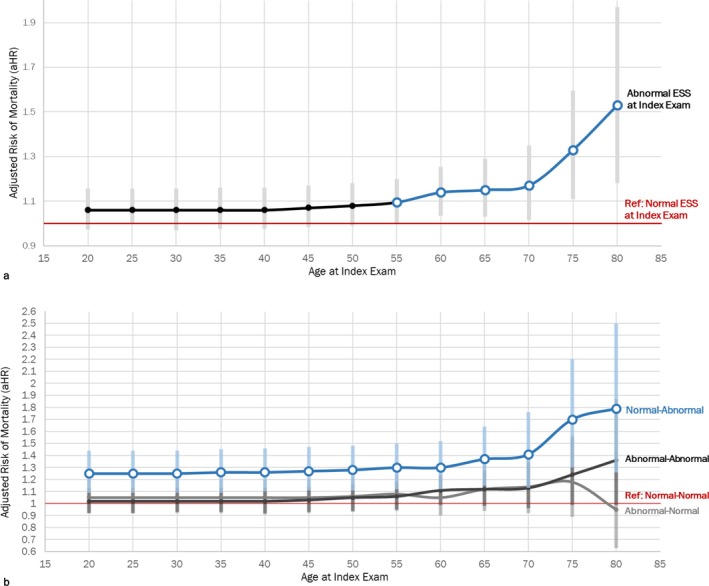
(a) The risk of adjusted 5‐year all‐cause mortality increased significantly in those with Abnormal ESS at their index exam in those 55 and older. (b) Those who converted from Normal‐Abnormal ESS had higher adjusted 5‐year mortality risk at all ages, with the greatest increase in risk after age 60. Elbow plot for the risk of 5‐year mortality across age ranges for (a) ESS at the index exam alone and (b) by ESS change status with 95% Confidence Intervals (grey), after adjustment for time between initial and index exam (days), age, sex, CCI, race, current CCI, history of OSA, PTSD and depression. Normal (a) and Normal‐Normal (b) ESS serve as the reference groups.

The relationship between age category and ESS was further elucidated in Figure 2. Veterans who were 55 and older with an Abnormal ESS at the index exam had a 10% higher adjusted risk of all‐cause mortality within 5 years when compared to those with a Normal ESS, regardless of ESS at the initial exam (*p* = 0.049; Figure 3a). Those who were Normal‐Abnormal had a significantly higher adjusted risk of all‐cause mortality within 5 years in all age categories compared to Normal‐Normal. However, among those with Normal‐Abnormal ESS, average adjusted risk was almost double in those over 55 (mean aHR: 1.47; aHR range: 1.30–1.77) when compared to Normal‐Normal (Figure 3b). Those with Abnormal‐Abnormal ESS and Abnormal‐Normal ESS did not have a significantly higher risk of all‐cause 5‐year mortality compared to Normal‐Normal ESS at any age.

**FIGURE 4:**
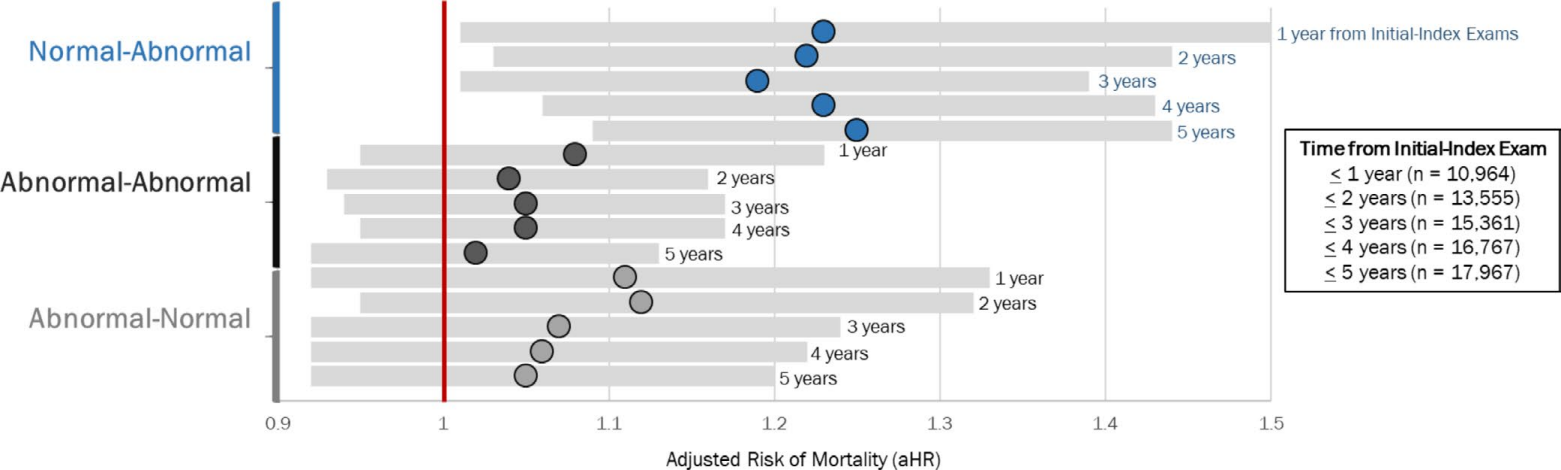
Those with Normal‐Abnormal ESS had a higher risk of 5‐year all‐cause mortality regardless of whether their initial exam was 1, 2, 3, 4 or 5 years before the index exam. Adjusted hazard ratios for 5‐year all‐cause mortality risk stratified by time between initial and index exams: 1 year or less (*n* = 10,964), 2 years or less (*n* = 13,555), 3 years or less (*n* = 15,361), 4 years or less (*n* = 16,767), 5 years or less (*n* = 17,967) for each ESS category: Normal‐Abnormal (blue), Abnormal‐Abnormal (black), Abnormal‐Normal (grey). Normal‐Normal ESS serves as the reference group (red vertical bar at 1.0).

Validation of the 5‐year threshold yielded that regardless of whether the initial and index exams were 1, 2, 3, 4 or 5 years apart, the adjusted risk of 5‐year all‐cause mortality was higher among those who were Normal‐Abnormal compared to Normal‐Normal (Figure 4). In contrast, being Abnormal‐Abnormal and Abnormal‐Normal was not associated with higher risk of death at any follow‐up period.

### 3.4 Comorbidities and ESS Change Groups

Univariate odds of identifying with Normal‐Abnormal ESS change status and 18 different comorbidities were evaluated (Table S1). Normal‐Abnormal Veterans also had higher unadjusted odds of incident chronic pulmonary disease (OR: 1.35; 95% CI: 1.12, 1.62) or experiencing an increase in BMI to over 35 kg/m^2^ (OR: 1.33; 95% CI: 1.11, 1.60) compared to other ESS change categories.

## 4 Discussion

In this retrospective study of 17,967 US Veteran adults with two sleep clinic encounters, development of EDS was associated with increased risk of all‐cause mortality within 5 years after adjustment for follow‐up time, age, sex, race and comorbid conditions. Risk was particularly high among Veterans with EDS who were 55 years of age and older and those who developed EDS between their initial and index exams.

### 4.1 Clinical Implications

These findings emphasise the burden of excessive sleepiness among Veterans seeking sleep services, where over half suffered from EDS at the initial exam, compared to 25% in the general population (Borsini et al. 2019). This work corroborates our previous finding that EDS is associated with a higher risk of adjusted all‐cause mortality in Veterans who are 60 and older (Maghsoudi et al. 2024). This longitudinal analysis was able to further identify that the risk was highest among those with emergent EDS (Normal‐Abnormal) compared to Normal‐Normal, after adjustment. Prior studies also support the complex relationship between sleep stability and mortality risk. In a large prospective study of adult twins, Hublin et al. found that changes in sleep length and sleep quality across a 6‐year period were associated with increased mortality risk (Hublin et al. 2007).

This work indicates that ESS may serve as a parsimonious marker for EDS‐associated comorbid conditions associated with 5‐year mortality risk after adjusting for follow‐up time, age, sex, race and comorbid conditions. Furthermore, these results emphasise the clinical impact of monitoring ESS changes over time, particularly among adults over the age of 55. Consistent with work conducted by Maghsoudi et al., Veterans over the age of 55 with a single Abnormal ESS may have a higher adjusted risk of 5‐year mortality compared to those with Normal ESS. Moreover, VA providers detecting negative changes across more than one ESS, from Normal‐Abnormal, may now better identify patients at elevated risk for mortality, particularly among older Veterans (Figure 3b). Specifically, two sequential ESS values that change from normal to abnormal within a 12‐week to 5‐year period may be an indication of comorbid conditions that could increase mortality risk compared to Normal‐Normal ESS. Although the time between initial and index exams ranged from 1 to 5 years, the median follow‐up time was less than 1 year. These results suggest that the adjusted risk of 5‐year all‐cause mortality is still higher among patients with Normal‐Abnormal ESS whether their initial exam was 6 or 60 months before their index exam.

Unexpectedly, persistent EDS (Abnormal‐Abnormal) and improvement from abnormal to normal (Abnormal‐Normal) did not confer different mortality risk compared to consistently normal sleepiness (Normal‐Normal). Several mechanisms may explain these findings. Veterans with EDS at the initial exam (Abnormal‐) may have received more intensive clinical surveillance, targeted treatments or management of underlying conditions, potentially mitigating excess mortality risk. Additionally, survivorship bias may exist such that individuals with severe comorbidities at initial presentation may not have survived to the second exam; therefore, the eligible sample may represent a healthier patient population despite ongoing symptoms. Conversely, veterans whose sleepiness improved (Abnormal‐Normal) may not have fully resolved underlying systemic risks, such as metabolic, cardiovascular or inflammatory conditions, that continued to influence mortality despite improved EDS symptoms. These findings underscore the need for longitudinal research evaluating how treatments and comorbidity progression may interact with changes in sleepiness and mortality risk.

### 4.2 Potential Mechanisms

Several potential explanatory mechanisms exist for the relationship between comorbid conditions associated with emergent EDS (Normal‐Abnormal) and 5‐year all‐cause mortality risk. Sleep disorders and sleep deficiency have been linked to many chronic medical conditions that may increase mortality risk, including lung disease, cardiovascular disease, diabetes, obesity, psychiatric illness, cancer, chronic renal disease, fibromyalgia, certain infectious diseases and cancer (Liew and Aung 2021; Laposky et al. 2016; Parish 2009). These conditions have the potential to increase EDS in a variety of ways, either by reducing nighttime sleep quality or requiring more restorative sleep throughout the day, increasing overall daytime sleepiness.

Among various comorbidities, including all 17 components of the Charlson‐Deyo Comorbidity Index and high BMI increases, only incident Chronic Pulmonary Disease and increases of BMI from under to over 35 kg/m^2^ were significantly associated with a higher probability of having a Normal‐Abnormal ESS (Table S1) in the present study. Chronic pulmonary diseases, such as chronic obstructive pulmonary disease (COPD) can lead to EDS by causing shortness of breath and reduced blood oxygen levels during sleep, disrupting sleep patterns and reducing sleep quality. COPD can also cause systemic inflammation and metabolic changes that may disrupt normal sleep cycles and increase the probability of EDS (Parish 2009; Del Brutto et al. 2024).

Similarly, chronic sleep deprivation and sleep disorders may also disrupt glucose metabolism, insulin sensitivity, appetite regulation and energy balance (Knutson et al. 2007; Liu et al. 2022). In a prospective study of over 1400 individuals, Brutto et al. identified that those with poor sleep quality had significantly less physical activity and higher fasting glucose than those with good sleep quality, which also increased mortality risk after adjustment (Del Brutto et al. 2024). Veterans who had a BMI under 35 kg/m^2^ at their initial exam but increased to 35 kg/m^2^ or more at the index exam had 33% higher odds of having Normal‐Abnormal ESS than those whose BMI did not increase over 35 kg/m^2^ (Table S1; OR = 1.33; 95% CI: 1.11, 1.60). Despite the relationship between metabolic disturbances and poor sleep with mortality, BMI at the index exam did not significantly improve model fit. Prior research suggests that EDS may be a consequence of obesity‐associated metabolic conditions rather than the cause, negatively impacting sleep quality and increasing the probability of EDS (Fernandez‐Mendoza et al. 2015; Vgontzas et al. 2014). Future research is needed to better understand the temporal relationship between these associated conditions and their longitudinal impact on mortality risk.

Other potential mechanisms exist but did not have statistically significant associations with Normal‐Abnormal ESS changes in the present study. Incident Diabetes Mellitus (DM), often co‐occurring with increases in BMI and associated with EDS‐associated glucose metabolism, was not associated with higher odds of having a Normal‐Abnormal ESS in the present study after Bonferroni correction (*p* > 0.003) (Nguyen et al. 2011). BMI increases often precede DM diagnoses and future work with a longer follow‐up time and a more targeted outcome evaluation is required to better understand the potential interplay between these conditions and EDS (Felber and Golay 2002). Additionally, sleep disorders, such as obstructive sleep apnea, are also known to increase sympathetic activity, systemic inflammation, oxidative stress and endothelial dysfunction that increase the risk for cardiovascular diseases (Mitra et al. 2021). Although the odds of incident OSA, MI or CHF and Normal‐Abnormal ESS were not statistically significant compared to other ESS changes (Table S1; OR = 1.24; 95% CI: 0.99, 1.54), this work lays the foundation for employing more targeted criteria to understand potential relationships between ESS changes and sleep‐disorder‐adjacent comorbidities. Finally, sleep disturbances are also associated with impaired immune and hormone regulation, such as increased circulating inflammatory cytokines, suppressed anti‐inflammatory markers and increased stress‐related hormone secretion (Vgontzas et al. 2014; Ditmer et al. 2021). Subsequent chronic inflammation is associated with cardiovascular disease, cancer and neurodegenerative disorders linked to higher mortality risk (Tao et al. 2021; Garbarino et al. 2021). However, these markers were not available in the present study, and more research is needed to understand the direction of the relationship between inflammation, EDS and mortality.

### 4.3 Strengths and Limitations

This study has many strengths. First, the novel method used to extract ESS values from sleep clinic notes makes this the first and largest study to evaluate longitudinal changes in EDS. The NLP extraction was performed with high accuracy, and the predictive ability of the final Cox proportional hazards model was very good (Harrell's C = 0.784) (Maghsoudi et al. 2024; Fibrinogen Studies Collaboration 2009). Second, the dynamic relationship between clinical exams and corresponding ESS measures provides a glimpse into the temporal relationship between 5‐year mortality risk and ESS category changes over time. Studies suggest that older Veterans, particularly those with cognitive impairment, may underreport EDS symptoms; therefore, the decision to evaluate categorical shifts in ESS (Normal vs. Abnormal) conservatively buffers for greater variation in ESS among older adults (Onen et al. 2013; Huang et al. 2024). Likewise, robust inclusion of age and follow‐up time as continuous variables in the full model, restriction to 5‐year follow‐up, and investigations of age‐stratified variation in ESS change and mortality attempted to minimise residual confounding from physical and mental health conditions more prevalent in ageing Veterans. Finally, the double‐dichotomisation analytic approach facilitates a practical clinical application of these results. Future work will build upon this research to understand the impact of the magnitude of change in continuous ESS values to define potential thresholds for mortality and disease risk among Veterans.

Some limitations exist. Although a validated measure in Veteran populations, the ESS is a subjective measure of self‐reported sleepiness and must be considered within the appropriate context without objective daytime assessment. Several prior studies question the reliability and reproducibility of the ESS, particularly among individuals with sleep‐related breathing (Walker et al. 2020; Nguyen et al. 2006; Taylor et al. 2019; Campbell et al. 2018; Lee et al. 2020). Conservative restrictions were employed to minimise potential confounding from clinical covariates that may be associated with EDS and mortality, such as a prior diagnosis of depression, PTSD and OSA, but may underestimate the prevalence of these conditions overall. For example, Goldstein et al. estimate that 21% of Veterans are diagnosed with OSA while this study identified 18.6% by the index exam (Goldstein et al. 2024). Likewise, smoking status was not available for most participants, with 87.0% undocumented at the time of either qualifying exam while over 50% are estimated to have smoked over 100 cigarettes in their lifetime (Mshigeni et al. 2021). These variables are described due to their historical associations with mortality; however, missing values were not imputed nor attributed post hoc to avoid differential information bias.

Results were limited to Veterans referred to sleep clinics within the VHA who were healthy and mobile enough to attend two exams at least 72 days apart. Moreover, Veterans who use VHA resources are older, are of lower socioeconomic status and education, are more likely to be African American, and are in poorer health than Veterans who seek care outside the VHA (Agha et al. 2000). Therefore, findings should not be generalised to the general population. Neither cause of death data, ESS in Veterans administered outside of VA sleep clinic services, nor treatments administered at the initial exam that may have influenced or improved ESS in the cohort were available for analysis. However, the CCI is a validated and widely used score within VHA to characterise a wide range of comorbidities and was available for all Veterans in the final model.

## 5 Conclusion

ESS can efficiently identify EDS, which may serve as a marker for mortality risk, particularly for Veterans aged 55 and older. Among Veterans seeking sleep services within VHA, change in ESS from normal to abnormal was associated with 5‐year all‐cause mortality in the context of age, sex, race and comorbid conditions. Veterans who experienced a change from Abnormal to Normal experienced the same 5‐year mortality risk as those who were persistently normal. Future research should evaluate specific causes of death associated with worsening EDS and which treatments administered mitigate risk of death associated with ESS.

## Author Contributions

**Katherine G. Bay:** conceptualization, investigation, writing – original draft, methodology, writing – review and editing, visualization, validation, formal analysis, project administration. **Arash Maghsoudi:** data curation, software, validation, project administration, supervision, resources, writing – review and editing, investigation. **Amin Ramezani:** data curation, software, validation. **Drew A. Helmer:** writing – review and editing. **Amir Sharafkhaneh:** funding acquisition, conceptualization, writing – review and editing, project administration, supervision, methodology, resources. **Javad Razjouyan:** funding acquisition, investigation, methodology, writing – review and editing, project administration, supervision, resources, data curation, software, conceptualization.

## Conflicts of Interest

The authors declare no conflicts of interest.

## Supporting information


**Table S1:** Incident comorbidities and odds of Normal‐Abnormal ESS changes between initial and index exams (*N* = 17,967).

## Data Availability

The data that support the findings of this study are available on request from the corresponding author. The data are not publicly available due to privacy or ethical restrictions.
