# Supplementary material for: Changes in Self‐Reported Excessive Daytime Sleepiness Are Associated With 5‐Year All‐Cause Mortality Risk Among Veterans
Source: J Sleep Res. 2025 Aug 19;35(2):e70168. doi: 10.1111/jsr.70168 (PMC12496456; doi:10.1111/jsr.70168)
Supplement: Supplementary file 1 — Table S1: Incident comorbidities and odds of Normal‐Abnormal ESS changes between initial and index exams (N = 17,967). [file JSR-35-e70168-s001.docx]

**Supplementary Table 1.** Incident Comorbidities and Odds of Normal-Abnormal ESS Changes between Initial and Index Exams (N = 17,967).

|  |  | **Normal-Normal n = 6,819 (37.95%)** | **Normal-Abnormal n = 1,806 (10.05%)** | **Abnormal-Abnormal n = 7,110 (39.57%)** | **Abnormal-Normal n = 2,232 (12.42%)** |  |  |
| --- | --- | --- | --- | --- | --- | --- | --- |
| **Categorical Variables** | **N (%)** | **N (%)** | **N (%)** | **N (%)** | **N (%)** | **OR (95% CI)** | **p-value** |
| AIDS | 3 (0.02%) | 1 (0.01%) | 1 (0.06%) | 1 (0.01%) | 0 (0.00%) | 4.48 (0.08, 86.01) | 0.180 |
| Any malignancy | 371 (2.06%) | 132 (1.94%) | 38 (2.09%) | 138 (1.94%) | 63 (2.82%) | 1.02 (0.71, 1.44) | 0.902 |
| BMI increase to >35 kg/m^2^ | 1,194 (6.65%) | 428 (6.28%) | 152 (8.42%) | 454 (6.39%) | 160 (7.17%) | 1.33 (1.11, 1.60) | **0.001** |
| Cerebrovascular disease | 364 (2.03%) | 134 (1.97%) | 46 (2.55%) | 133 (1.87%) | 51 (2.55%) | 1.30 (0.93, 1.79) | 0.097 |
| Chronic pulmonary disease | 1,130 (6.29%) | 371 (5.44%) | 145 (8.03%) | 436 (6.13%) | 178 (7.97%) | 1.35 (1.12, 1.62) | **0.001** |
| Congestive heart failure | 521 (2.90%) | 207 (3.04%) | 63 (3.47%) | 169 (2.38%) | 82 (3.67%) | 1.24 (0.93, 1.62) | 0.116 |
| Dementia | 90 (0.50%) | 43 (0.63%) | 10 (0.55%) | 22 (0.31%) | 15 (0.67%) | 1.12 (0.52, 2.17) | 0.738 |
| Diabetes with complications | 569 (3.17%) | 217 (3.18%) | 58 (3.21%) | 215 (3.02%) | 79 (3.54%) | 1.02 (0.76, 1.34) | 0.909 |
| Diabetes without complications | 647 (3.60%) | 225 (3.30%) | 75 (4.15%) | 259 (3.64%) | 88 (3.94%) | 1.18 (.91, 1.51) | 0.185 |
| Hemiplegia or paraplegia | 26 (0.14%) | 13 (0.19%) | 3 (0.17%) | 7 (0.10%) | 3 (0.13%) | 1.17 (0.22, 3.87) | 0.801 |
| Metastatic solid tumor | 38 (0.21%) | 18 (0.26%) | 2 (0.11%) | 9 (0.13%) | 9 (0.40%) | 0.5 (0.06, 1.93) | 0.326 |
| Mild liver disease | 203 (1.13%) | 73 (1.07%) | 18 (1.00%) | 72 (1.01%) | 40 (1.79%) | 0.87 (0.50, 1.42) | 0.572 |
| Moderate/severe liver disease | 25 (0.14%) | 10 (0.15%) | 4 (0.22%) | 5 (0.07%) | 6 (0.27%) | 1.71 (0.43, 5.06) | 0.322 |
| Myocardial infarction | 277 (1.54%) | 108 (1.58%) | 33 (1.83%) | 91 (1.28%) | 45 (2.02%) | 1.21 (0.82, 1.76) | 0.299 |
| Obstructive Sleep Apnea | 456 (2.54%) | 168 (2.46%) | 42 (2.3%) | 181 (2.55%) | 65 (2.91%) | 0.91 (0.64, 1.25) | 0.545 |
| Peptic ulcer disease | 90 (0.50%) | 37 (0.54%) | 7 (0.39%) | 30 (0.42%) | 16 (0.72%) | 0.75 (0.29, 1.63) | 0.472 |
| Peripheral vascular disease | 421 (2.34%) | 158 (2.32%) | 48 (2.66%) | 154 (2.17%) | 61 (2.73%) | 1.16 (0.83, 1.57) | 0.351 |
| Renal disease | 416 (2.32%) | 162 (2.38%) | 41 (2.27%) | 151 (2.12%) | 62 (2.78%) | 0.98 (.69, 1.36) | 0.893 |
| Rheumatoid disease | 71 (0.40%) | 27 (0.40%) | 6 (0.33%) | 24 (0.34%) | 14 (0.63%) | 0.83 (0.29, 1.90) | 0.653 |

*Represents whether Veterans presented with comorbidities at the Index exam but not the initial exam, stratified by ESS change status. Odds ratios, confidence interval, and p-values reflect odds of Normal-Abnormal (compared to all other ESS change categories) co-occurring with each incident comorbid condition. Significance level considered with α=0.05 after Bonferroni correction = 0.003.*
